# Spectrum of clinical disease in a series of 135 hospitalised HIV-infected patients from north India

**DOI:** 10.1186/1471-2334-4-52

**Published:** 2004-11-22

**Authors:** SK Sharma, Tamilarasu Kadhiravan, Amit Banga, Tarun Goyal, Indrish Bhatia, PK Saha

**Affiliations:** 1 Department of Medicine, All India Institute of Medical Sciences, New Delhi, India

## Abstract

**Background:**

Literature on the spectrum of opportunistic disease in human immunodeficiency virus (HIV)-infected patients from developing countries is sparse. The objective of this study was to document the spectrum and determine the frequency of various opportunistic infections (OIs) and non-infectious opportunistic diseases, in hospitalised HIV-infected patients from north India.

**Methods:**

One hundred and thirty five consecutive, HIV-infected patients (age 34 ± 10 years, females 17%) admitted to a tertiary care hospital in north India, for the evaluation and management of an OI or HIV-related disorder between January 2000 and July 2003, were studied.

**Results:**

Fever (71%) and weight loss (65%) were the commonest presenting symptoms. Heterosexual transmission was the commonest mode of HIV-acquisition. Tuberculosis (TB) was the commonest OI (71%) followed by candidiasis (39.3%), *Pneumocystis jiroveci *pneumonia (PCP) (7.4%), cryptococcal meningitis and cerebral toxoplasmosis (3.7% each). Most of the cases of TB were disseminated (64%). Apart from other well-recognised OIs, two patients had visceral leishmaniasis. Two cases of HIV-associated lymphoma were encountered. CD4+ cell counts were done in 109 patients. Majority of the patients (82.6%) had CD4+ counts <200 cells/μL. Fifty patients (46%) had CD4+ counts <50 cells/μL. Only 50 patients (37%) received antiretroviral therapy. Twenty one patients (16%) died during hospital stay. All but one deaths were due to TB (16 patients; 76%) and PCP (4 patients; 19%).

**Conclusions:**

A wide spectrum of disease, including both OIs and non-infectious opportunistic diseases, is seen in hospitalised HIV-infected patients from north India. Tuberculosis remains the most common OI and is the commonest cause of death in these patients.

## Background

The first case of human immunodeficiency virus/acquired immunodeficiency syndrome (HIV/AIDS) in India was detected in 1986 in the state of Tamilnadu [1] and since then the spread of HIV/AIDS across the nation has been relentless. Cases have been reported from all states and union territories of India. Though the overall prevalence of HIV infection is low (<1%), the total number of cases is high. As per estimates, by the end of 2002, about 4.58 million adults were infected with HIV [2]. Even more ominous has been the shift of the epidemic from high-risk groups such as injectable drug users (IDU) and patients with other sexually transmitted diseases, to low-risk groups like married, monogamous women [3].

Though the majority of HIV-infected population lives in developing nations, there is a paucity of data on natural history, pattern of disease and survival of hospitalised patients with HIV/AIDS from these regions, especially India. It is well established that manifestations of AIDS are influenced by factors such as endemic infections and malnutrition that are widely prevalent in these regions [4]. Conventional disease staging criteria, which were developed in western populations may not hold good in these settings [4,5]. Added to this, resource constraints prohibit evaluation and decision-making based on cost and labour-intensive methods such as CD4+ cell counts and viral RNA load estimation. Timely initiation of prophylaxis for opportunistic infections (OIs) and their prompt recognition and treatment are the only economically viable options [6]. In this scenario, knowledge regarding the pattern of OIs, will be useful. This study was conducted to elucidate the frequency of various OIs and non-infectious opportunistic conditions in hospitalised HIV-infected patients, from north India.

## Methods

We describe a series of 135 consecutive patients with HIV/AIDS, aged 13 years and above, admitted to the All India Institute of Medical Sciences (A.I.I.M.S.) hospital, New Delhi during the period of January 2000 through July 2003. A.I.I.M.S. is a large tertiary level teaching hospital and referral centre located in north India, attending to HIV-infected patients apart from others. The catchment area of this hospital includes the New Delhi national capital territory and neighbouring states mainly, Uttar Pradesh, Bihar, Uttharanchal, Haryana, Punjab and Himachal Pradesh. All patients were under the care of a single unit, of the three units existing in the Department of Medicine. Decision to admit was taken by the treating physician and all patients were hospitalised for the evaluation and treatment of a suspected OI or HIV-related disorder. We have earlier reported the determinants of in-hospital mortality in this series [7].

Diagnosis of HIV infection was made using methods described previously [8]. All patients referred to A.I.I.M.S. as 'HIV-positive' underwent retesting. Apart from this, all patients treated for tuberculosis (TB), those reporting risk factors for HIV infection and patients with clinical presentation suggestive of underlying immunosuppression, were offered HIV testing. All except one patient were infected with HIV-1, the latter with HIV-2. CD4+ lymphocyte counts were done by flowcytometry, using a FACSCalibur flowcytometer (Becton Dickinson, USA) in patients who could afford for the same. Patients were evaluated using a predesigned instrument regarding the demographic characteristics, risk factors for HIV infection, presenting symptoms, physical findings and laboratory parameters.

Various laboratory abnormalities were defined using cut-off values as follows: anaemia – haemoglobin <100 g/L; leucopenia – total leucocyte count (TLC) <4 × 10^9^/L; lymphocytopenia – absolute lymphocyte count (ALC) <1.2 × 10^9^/L; decreased CD4+ – <500 cells/μL; thrombocytopenia – platelet count <150 × 10^9^/L; elevated erythrocyte sedimentation rate (ESR) – >20 in the first hour; hypoalbuminaemia – albumin <35 g/L; hyperglobulinaemia – globulin >40 g/L; hyperbilirubinaemia – serum total bilirubin >20.5 μmol/L; elevated liver enzymes – >2 times the upper limits of normal [aspartate aminotransferase (AST) – >100 IU/L, alanine aminotransferase (ALT) – >100 IU/L, alkaline phosphatase (ALP) – >560 IU/L]; hypoxaemia – PaO_2 _<12 kPa; increased arterial-alveolar oxygen gradient (A-a DO_2_) – >3.3 kPa, while breathing ambient air (F_i_O_2 _= 0.21).

While genuine efforts were made to establish the diagnosis of an OI, patients at times were too sick to undergo invasive diagnostic procedures such as bronchoscopy or tissue biopsy and hence the following criteria were used to define an OI concerned. Pulmonary tuberculosis (PTB): clinical features suggestive of TB with radiological features compatible with TB on chest radiograph or computed tomographic scan (CT) and/or demonstration of acid-fast bacilli (AFB) in sputum smears or growth of *Mycobacterium tuberculosis *in sputum culture; disseminated tuberculosis (DTB): clinical features suggestive of TB with concurrent involvement of at least two non-contiguous organs, in the presence of bacteriological and/or histopathological evidence of TB and improvement with anti-tuberculosis therapy; miliary tuberculosis (MTB): clinical presentation consistent with TB and bacteriological and/or histopathological evidence of TB and demonstration of bilateral miliary infiltrates on chest radiograph or high-resolution CT; tuberculosis meningitis (TBM): clinical and cerebrospinal fluid (CSF) features consistent with tuberculosis meningitis, with bacteriologic demonstration of *M. tuberculosis *in CSF or after exclusion of other aetiologies such as cryptococcus and syphilis, with evidence of tuberculosis elsewhere and/or improvement with anti-tuberculosis therapy; cryptococcal meningitis: in CSF, demonstration of *Cryptococcus *sp. yeast cells by India ink or antigen by latex agglutination or growth in culture; cerebral toxoplasmosis: demonstration of multiple ring-enhancing cerebral parenchymal lesions on contrast-enhanced CT or magnetic resonance imaging (MRI), in the presence of anti-toxoplasma antibody in serum and clinical response to anti-toxoplasma therapy; progressive multifocal leucoencephalopathy (PMLE): compatible clinical presentation with demonstration of characteristic cerebral white matter changes by MRI; *Pneumocystis jiroveci *pneumonia (PCP): bilateral, diffuse interstitial infiltrates on chest radiograph or high-resolution CT, with hypoxaemia (PaO_2 _<12 kPa) and sputum smears/cultures negative for aerobic bacteria and AFB and/or demonstration of *Pneumocystis jiroveci *in induced sputum [9].

Patients received primary chemoprophylaxis for PCP and toxoplasmosis and received treatment for specific OIs followed by secondary prophylaxis, as per recommendations [10]. For patients with PCP, whenever hypoxaemia was severe (PaO_2 _<9.3 kPa), corticosteroids were given in addition to oral co-trimoxazole. None of these patients received assisted ventilation. All patients were counselled and offered antiretroviral therapy, whenever indicated. Upon discharge from the hospital, patients were advised to follow-up as outpatients, on a regular and as needed basis.

### Statistical analysis

Entry and analysis of all data were done using a statistical software package (SPSS for Windows, Version 10.0, SPSS Inc., Chicago, IL). Entered data were double-checked for discrepancies. Data are presented as mean ± standard deviation (SD) when distributed normally and as median with interquartile range (IQR), if the distribution was skewed. Frequency of various clinical and laboratory findings and the frequencies of individual OIs are expressed as proportions (%). The relation between ALC and CD4+ count was analysed by Pearson's product moment correlation. Paired-samples t-test was applied to assess the improvement in CD4+ cell counts following antiretroviral therapy.

## Results

Over a period of about three and a half years, 135 patients with HIV/AIDS were admitted and all these patients were included in the study. Mean age of the study group was 34 ± 10 (range: 13–73) years. Females constituted less than one fourth of the study group (17%). Details of other demographic characteristics and transmission categories are presented in Table-1. More than two-thirds of patients (76%) were in the second and third decades of their lives. Labourers were the commonest occupational group (40%) and long-haul truck drivers constituted a considerable proportion of the study group. Professionals were the least common occupational group. An identifiable risk factor for HIV infection was present in 59% of patients, extramarital heterosexual contact being the commonest (41.5%). None of the patients reported homosexual practices. About 40% of patients denied any risk factor for the acquisition of HIV infection. A large number of patients (44.4%) were from states other than Delhi.

**Figure 1:**
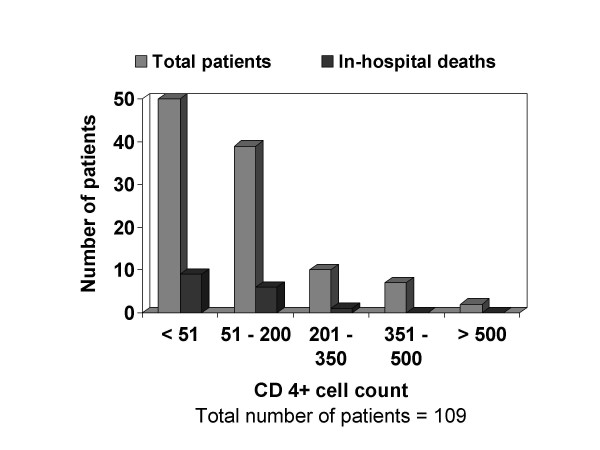
Distribution of CD4+ cell counts and in-hospital deaths in the respective groups

**Figure 2:**
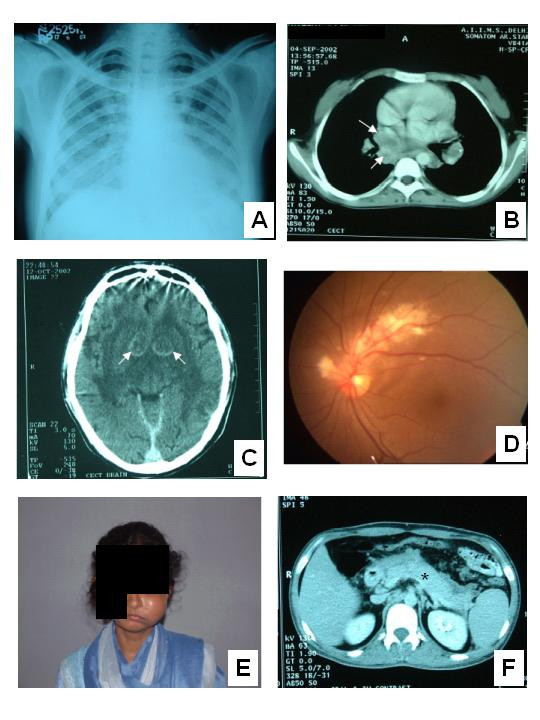
A – Chest radiograph of a patient with *Pneumocystis jiroveci *pneumonia showing bilateral, diffuse interstitial infiltrates B – Contrast enhanced computed tomographic (CT) scan of chest showing mediastinal lymphadenopathy in a patient with disseminated tuberculosis. Typical central necrosis evident as low attenuation areas (arrows) is seen C – Contrast enhanced CT scan of brain showing ring enhancing lesions in the basal ganglia bilaterally (arrows). Serology was positive for toxoplasma infection D – Ophthalmoscopic image of a patient with cytomegalovirus retinitis E – Non-Hodgkin's lymphoma in a HIV-infected lady presenting as unilateral maxillary swelling F – Contrast enhanced CT scan of abdomen reveals an oedematous and enlarged pancreas (asterisk) suggestive of acute pancreatitis. The patient was on didanosine and improved following withdrawal of the same and supportive treatment.

Three symptoms namely fever, weight loss and diarrhoea were the common symptoms at presentation (70.4%, 65.2% and 23.7% respectively). Productive cough and dyspnoea were present in about a fourth of patients (Table-2). Two-thirds of patients (66.7%) were malnourished (body mass index <19 kg/m^2^) and generalised lymphadenopathy was present in a considerable proportion of patients (16.3%). Poor performance on mini mental status examination (MMSE score <23) was not uncommon (20.7%). Altered sensorium and focal neurologic deficit were encountered occasionally. Anaemia was present in about half of the patients (50.5%) and among those who where anaemic, 17 (21.8%) patients were on zidovudine. A considerable number of patients were leucopenic and in 22% of patients ALC was less than 1200/μL (Table-3). CD4+ cell counts were done in 109 patients. The distribution of CD4+ cell counts is shown in Figure-1. Most of the patients (n = 90; 82.6%) had CD4+ counts less than 200 cells/μL. Fifty patients (46%) had CD4+ counts less than 50 cells/μL. The correlation of ALC with CD4+ count was not significant (r = 0.14; P = 0.18). HIV viral load estimation was done in only four patients (range 33752–289176 RNA copies/mL). Twenty patients (14.8%) had hypoxaemia. Of these, five patients had PCP (Figure 2-A), ten had DTB, three had extensive PTB and one patient had massive unilateral tuberculosis pleural effusion.

The mean number of OI was 1.4 per patient. The commonest OI was TB (71.1%), followed by oral candidiasis (39.3%). Most of the cases of TB were disseminated (64%; Figure 2-B) and PTB was seen in only a small number of patients (8.1%). Eleven patients had MTB (Table-4). Of the 53 patients with oral candidiasis, five patients had concomitant oesophageal involvement. Cryptosporidium sp. and Giardia sp. infection causing diarrhoea was found in three and two patients respectively. Of the 13 cases of meningitis, eight were tuberculosis and the rest due to Cryptococcus sp.; no case of acute, pyogenic meningitis was seen. Cerebral toxoplasmosis (Figure 2-C) and one case of PMLE were the other central nervous system infections seen.

Of the five cases of cerebral toxoplasmosis, one patient presented as recent onset dilated cardiomyopathy with no features to suggest a central nervous system pathology. On further investigation, the patient was found to be seropositive for anti-toxoplasma antibodies and contrast enhanced CT revealed multiple ring enhancing cerebral lesions. The cerebral lesions cleared and cardiac ejection fraction improved with anti-toxoplasma treatment. The cardiac dysfunction was probably due to toxoplasma myocarditis. Ten patients presented with PCP (Figure 2-A) and three patients had cytomegalovirus (CMV) retinitis (Figure 2-D). One patient was diagnosed as disseminated histoplasmosis. This patient presented as fever of unknown origin. Bone marrow biopsy revealed caseating granulomas and subsequently fungal culture grew *Histoplasma capsulatum*. Non-infectious AIDS-defining conditions were not common. One case of B-cell non-Hodgkin's lymphoma presenting as unilateral maxillary swelling (Figure 2-E) and another case of Hodgkin's lymphoma that presented as generalised lymphadenopathy with hepatosplenomegaly, were seen.

Only 50 patients (37%) received antiretroviral therapy, of which three patients were taking protease inhibitor (PI) based regimens. All other patients received non-nucleoside reverse transcriptase inhibitor (NNRTI) based (nevirapine – 46 patients, efavirenz – one patient) triple-drug regimens. Follow-up CD4+ counts were done in 16 patients after three to six months of antiretroviral therapy. The mean CD4+ count increased to 224 ± 178 cells/μL from a baseline mean of 131 ± 122 cells/μL, which was statistically significant (P = 0.003). Myelosuppression during antiretroviral therapy developed in four patients, who were taking regimens containing zidovudine. One patient who was on didanosine developed acute pancreatitis (Figure 2-F). Another patient presented with severe, uncompensated, wide anion-gap metabolic acidosis with no apparent cause. This was probably due to lactic acidosis caused by stavudine, which he had been taking.

Twenty one patients (15.6%) died in hospital, most of them due to TB (16 patients) and PCP (4 patients). All patients who died in hospital except for one, had CD4+ counts less than 200 cells/μL (Figure-1).

## Discussion

Opportunistic infections (OIs) are the major cause of morbidity and mortality in patients with HIV infection. In resource-limited settings, knowledge regarding the prevalence of various OIs might aid in making decisions regarding empirical treatment and would help to prioritise limited resources. To this end, we studied the frequency of various opportunistic conditions in this series of 135 HIV-infected patients. Nearly a half of these patients came from places other than New Delhi, being referred by medical practitioners or approached on their own. In India, HIV-infected patients are usually treated in tertiary level centres, due to lack of expertise and facilities in primary and district level hospitals. Moreover, for reasons of privacy patients and their kin prefer treatment at places far off from their home towns. This series comprises predominantly of patients from the northern states of India. Hence the findings of the present study may not apply to south India and north-east Indian states. Moreover, being a hospital based study, patients with severe illness causing rapid death and patients with milder symptoms who prefer treatment from local health facilities may not have been proportionately represented in the study group.

Most of the patients were in the age group 21–40 years and males were predominantly affected. This is similar to nation-level statistics in which, of the 57781 cases of HIV/AIDS reported to the National AIDS Control Organisation (NACO), 89% of the cases were in the age group 15–44 years and 74% were males [11]. This section of the population is more affected because they are sexually more active and the social structure is patriarchal. Unfortunately, these patients also happen to be in the economically most productive years of their lives. The male preponderance might have been due to the fact that in the existing social milieu, females do not seek medical care fearing ostracism and loss of family support. Interestingly, a large number of patients (41%) did not give a history of any of the known risk factors for the acquisition of HIV infection and homosexual practice was singularly not seen. It is likely that these patients were reluctant to reveal, due to the prevailing social values, which discourage polygamy and homosexuality. For the same reason, despite a history of prior blood transfusion in a considerable proportion of patients, it may not be possible to ascribe causation; not all these patients would have acquired HIV infection through blood transfusion. Injectable drug users (IDU) constituted only a minority of the study group as has been observed in other parts of India except for the north-eastern states where IDU is widely prevalent [12].

The three symptoms, namely fever, weight loss and diarrhoea were common presenting features. In this series of patients, all of whom had some opportunistic condition at presentation indicating advanced HIV disease, wasting is anticipated. The association of severe weight loss at presentation with TB in HIV-infected patients has been reported before [13]. TB was very common in this series and was the commonest OI. Previous studies also confirm the high prevalence of TB in HIV-infected individuals in India [4,9,14,15]. Most of these cases of TB were either disseminated or extra-pulmonary. We have previously reported the increased prevalence of HIV seropositivity among cases of extrapulmonary TB as compared to PTB and as such, HIV co-infection is now on the rising trend among cases of TB, in this population [16]. This trend is in consonance with findings from across the globe. The cases of TB attributable to HIV are increasing and have reached alarming proportions in certain countries. An example of such a nation is the United States of America, where almost 26% of cases of TB are now attributable to HIV infection [17]. Although these figures are from countries where the incidence of TB has otherwise been low, it is bound to be similar in countries where TB is endemic. PCP was seen in only a small number of patients. This is in sharp contrast with western populations where PCP is the commonest AIDS-defining illness [18]. Earlier studies from India also have reported a low prevalence (6–7%) of PCP [9,14]. The low prevalence of PCP in developing countries is well known and the incidence is probably increasing [19].

The frequencies of various OIs as observed in the present study are similar to those reported earlier from other parts of India [4,9,14,15]. Some of the opportunistic conditions were conspicuous by their absence. Disseminated infection caused by the fungus *Penicillium marneffei *has been reported to be the third commonest OI in southeast Asia [20] and is known to be highly prevalent in the north-east Indian state, Manipur [21] which shares common geographic, ecologic and climatic conditions with the south-east Asian regions of *P. marneffei *endemicity. No case of *P. marneffei *infection was found in the present study. Kaposi's sarcoma, atypical mycobacterial infection and disseminated CMV disease were not seen, as has been observed by a previous study from south India [14]. It appears that, TB being an endemic infection in this population, takes its toll before other less virulent pathogens could come into play. Development of TB per se might have afforded some protection against atypical mycobacterial disease [22]. It is also possible that TB masked the recognition of other OIs, which might have been present since it is well known that a large number of potentially fatal OIs are not diagnosed antemortem [23,24]. In India, Kaposi's sarcoma occurring in the setting of HIV infection and other immunocompromised conditions is a rarity, though not undescribed [25-27]. As such the prevalence of human herpes virus-8 (HHV-8) infection among the healthy population in India is low when compared with African nations and this is also likely to be responsible for the rarity of Kaposi's sarcoma [28]. Apart from the well-recognised OIs, infection unique to the local population namely, visceral leishmaniasis occurs in association with HIV and this has to be borne in mind.

Overall in-hospital mortality in this series is considerable and is probably reflective of the advanced nature of disease at presentation. This is evidenced by the fact that more than 80% of patients had CD4+ counts less than 200 cells/μL and all but one deaths occurred in this group. However, as we had reported earlier, CD4+ cell counts do not determine the immediate outcome of patients with an OI, whereas the latter does [7]. The proximate causes of death in these patients are OIs, which are amenable to effective treatment. Many of these patients were from poor socioeconomic strata and were not able to afford for antiretroviral therapy. Cost constraints directly or indirectly, probably also resulted in poor compliance with scheduled follow-up visits. With the implementation of World Health Organisation sponsored '3 by 5' initiative, it is hoped that antiretroviral therapy will be within the reach for a large number these patients and also compliance is likely to become better.

## Conclusions

A wide spectrum of OIs is seen in hospitalised patients with HIV/AIDS from north India. Tuberculosis is the most common OI in this group of patients. Though uncommon, non-infectious opportunistic conditions also occur. Many patients suffer from more than one OI. Hospital mortality is significant, with TB and PCP being responsible for most of these deaths. Whenever a definite diagnosis is not established quickly, empirical treatment should be considered. Such an approach is likely to improve immediate outcome in hospitalised patients with HIV/AIDS.

## Competing interests

The author(s) declare that they have no competing interests.

## Authors' contributions

SKS conceived of the study, designed and coordinated the study and revised the manuscript critically for important intellectual content. TK participated in the design of the study and statistical analysis and drafted the manuscript. AB participated in the design of the study, performed the statistical analysis and revised the manuscript for important intellectual content. TG participated in the design of the study, collected the clinical data and contributed significantly to the content of the manuscript. IB participated in the design of the study, collected the clinical data and participated in drafting the manuscript. PKS participated in the design of the study and coordination and also contributed significantly to the content of the manuscript. All authors read and approved the final manuscript.

## Pre-publication history

The pre-publication history for this paper can be accessed here:



## Figures and Tables

**Table 1:** Demographic characteristics and transmission-risk categories in a series of 135 patients with HIV/AIDS

**Parameter**	**Number of patients, n (%)**
Age, in years (mean ± SD)	34 ± 10
Males	112(83)
Outside Delhi (n = 108)	48(44.4)
Occupation (n = 106)*	
Unskilled labourer	31(29.2)
Skilled labourer	11(10.4)
Driver	11(10.4)
Police and armed forces	9(8.5)
Businessmen	19(17.9)
Clerical	5(4.7)
Professional	2(1.9)
Others	18(17)
Risk categories	
Heterosexual contact	56(41.5)
IDU	5(3.7)
Blood transfusion	19(14.1)
Unknown^†^	55(40.7)

* 29 patients were unemployed; † Patients denied any of the known risk factors; IDU – Injectable drug users.

**Table 2:** Presenting features and physical findings in a series of 135 patients with HIV/AIDS

**Symptoms and signs**	Frequency* n (%)
Fever	95(70.4)
Weight loss	88(65.2)
Cough	57(42.2)
Expectoration	38(28.1)
Dyspnoea	34(25.2)
Diarrhoea	32(23.7)
Abdominal pain	31(23)
Vomiting	22(16.3)
Night sweats	19(14.1)
Chest pain	17(12.6)
Haemoptysis	4(3)
Altered sensorium	5(3.7)
Seizures	4(3)
Body mass index <19 kg/m^2 ^(n = 78)	52(66.7)
Midarm circumference^†^, in cm (mean ± SD) (n = 76)	18.9 ± 4.2
Triceps skin-fold thickness^†^, in mm (mean ± SD) (n = 78)	6.9 ± 1.7
Lymphadenopathy – cervical	39(28.9)
– axillary	32(23.7)
– mediastinal	18(13.3)
– retroperitoneal	15(11.1)
– generalised	22(16.3)
Hepatomegaly	29(21.5)
Splenomegaly	24(17.8)
Pleural effusion	15(11.1)
Ascites	3(2.2)
Pericardial effusion	3(2.2)
Jaundice	9(6.7)
MMSE score <23	28(20.7)
Nuchal rigidity	12(8.9)
Focal neurologic deficit	8(5.9)

* The denominator is 135, except for anthropometric parameters, where the number of patients is given in parentheses; † Measured at midpoint between acromian and olecranon processes of non-dominant arm: mean of three consecutive readings was taken; MMSE – Mini mental status examination.

**Table 3:** Laboratory findings in a series of 135 patients with HIV/AIDS

**Variable**	**Unit**	**Mean ± SD**	**Range**	Abnormality*%
Haemoglobin	g/L	98 ± 27	33 – 161	50.5
TLC	× 10^9^/L	6.2 ± 2.8	0.9 – 15.3	17.3
Platelet count	× 10^9^/L	267 ± 183	56 – 960	19.3
ESR	mm/hr	56 ± 18	12 – 91	96.2
ALC	× 10^9^/L	1.7 ± 0.8	0.1 – 3.6	22
CD4+ cell count (n = 109)	per μL	121 ± 205	0 – 1425	98.2
Serum albumin	g/L	30 ± 8	16 – 51	70.7
Serum globulin	g/L	45 ± 11	21 – 76	70.3
Serum bilirubin – total	μmol/L	18.8 ± 32.5	3.4 – 220.6	6.7
AST	IU/L	77 ± 113	2 – 790	17
ALT	IU/L	76 ± 92	2 – 682	22.3
ALP	IU/L	360 ± 419	44 – 2355	11.1
PaO_2 _(n = 31)	kPa	10.6 ± 3.7	4.6 – 19.4	14.8^†^
A-a DO_2 _(n = 31)	kPa	2.8 ± 2.4	0 – 7.5	7.4^†^

* Defined by cut-off values as mentioned in the text; † Denominator was taken as 135; TLC – Total leucocyte count; ALC – Absolute lymphocyte count; ESR – Erythrocyte sedimentation rate; AST – Alanine aminotransferase; ALT – Aspartate aminotransferase; ALP – Alkaline phosphatase; PaO_2 _– Partial pressure of oxygen; A-a DO_2 _– Arterial-alveolar oxygen gradient.

**Table 4:** Opportunistic infections and HIV-related conditions in a series of 135 patients with HIV/AIDS and in-hospital outcomes

**Diagnosis**	Frequency n*(%)	**CD4+ count median (IQR)**	**In-hospital deaths, n^†^(%)**
Tuberculosis – any organ	96(71.1)	46 (12–121)	16(16.7)
PTB	11(8.1)	46 (5–218)	3(27.3)
Pleural	4(3)	52 (5–276)	--
LNTB	1(0.7)	37^‡^	--
TBM	8(5.9)	58 (5–165)	1(12.5)
DTB	61(45.2)	47 (15–127)	11(18)
MTB	11(8.1)	27 (5–57)	1(9.1)
Candidiasis	53(39.3)	54 (12–77)	9(17)
PCP	10(7.4)	38 (8–69)	4(40)
Herpes Zoster	8(5.9)	91 (59–151)	1(12.5)
Cryptococcal meningitis	5(3.7)	135 (14–320)	2(40)
Toxoplasmosis	5(3.7)	72 (21–132)	1(20)
Cryptosporidiosis	3(2.2)	61 (5–116)	--
CMV retinitis	3(2.2)	127 (37–150)	1(33.3)
Giardiasis	2(1.5)	59 (1–116)	--
Visceral leishmaniasis	2(1.5)	41 (5–77)	--
Lymphoma	2(1.5)	145 (29–261)	1(50)
Disseminated histoplasmosis	1(0.7)	51^‡^	--
CMV oesophagitis	1(0.7)	8^‡^	--
PMLE	1(0.7)	62^‡^	--

* Not mutually exclusive; † Total will exceed the mortality of the study group; ‡ Single value; – No deaths; IQR – interquartile range; PTB – pulmonary tuberculosis; Pleural – tuberculosis pleural effusion; LNTB – lymph node tuberculosis; TBM – tuberculosis meningitis; DTB – disseminated tuberculosis; MTB – miliary tuberculosis; PCP – *Pneumocystis jiroveci *pneumonia; CMV – Cytomegalovirus; PMLE – progressive multifocal leucoencephalopathy.
